# Time-Course Analysis of Cognitive Benefits Following Self-Stretching Using a Self-Control Design: Evidence from Stroop, N-Back, and Memory Tasks

**DOI:** 10.3390/brainsci15121285

**Published:** 2025-11-29

**Authors:** Sang-Young Park, Se-Yeon Park, Seo-Yoon Park, Seunghue Oh

**Affiliations:** 1 Department of Physical Therapy, Uiduk University, Gyeongju 38004, Republic of Korea; sypark@uu.ac.kr (S.-Y.P.); arclain@naver.com (S.-Y.P.); 2 Department of Physical Therapy, College of Health and Welfare, Woosuk University, Wanju-gun 55338, Republic of Korea; pgy0614@hanmail.net

**Keywords:** self-stretching, cognitive function, attention, reaction time, memory, time-course

## Abstract

**Background:** While physical activity has been shown to enhance cognitive function, little is known about whether stretching, a low-intensity, accessible form of exercise, can elicit similar benefits, particularly over time. The study adopted a self-control (within-subject) design. **Purpose:** This study aimed to investigate the time-dependent effects of self-stretching on cognitive performance in healthy adults. **Methods:** Thirty healthy participants performed a self-stretching protocol targeting the neck and shoulder muscles. Cognitive performance was assessed in terms of attention (Stroop test), working memory (N-back test), and short-term memory (digit span-based memory test) at baseline, and at 10, 20, and 30 min post-intervention. Paired t-tests and Wilcoxon signed-rank tests were used to analyze changes in cognitive performance. One-way ANOVA was conducted on baseline data to confirm the homogeneity across the three measurement groups. **Results:** One-way ANOVAs confirmed no significant baseline differences among the three groups in any cognitive measure (*p* > 0.05). Stroop test results showed a significant reduction in reaction time at 10 (*p* = 0.01) and 20 min (*p* = 0.02) post-stretching, indicating an improvement in information processing speed. The effect size (Hedges’ g) for this improvement was very large (−1.01) at 10 min and large (−0.87) at 20 min. However, no significant improvements were observed in Stroop accuracy scores, memory performance, or N-back task results at any time point. **Conclusions:** Self-stretching produced short-term improvements only in reaction time during attention tasks, while no changes were observed in memory or working memory accuracy. These findings suggest that stretching may offer brief, task-specific benefits related to processing speed rather than broad cognitive enhancement.

## 1. Introduction

Enhanced cognitive function, such as attention, memory, and processing speed, plays a crucial role not only in knowledge acquisition but also in motor learning, thereby facilitating both academic and physical skill development [1,2,3,4,5]. In clinical settings, cognition is a key contributor to the effectiveness of rehabilitation, where improvements in attention and executive function can positively influence motivation, compliance, and recovery outcomes [6,7]. Thus, strengthening cognitive performance holds cross-disciplinary value in education, sports, and therapeutic contexts.

A growing body of evidence supports the beneficial effects of physical activity on cognitive function [8]. In particular, acute bouts of moderate-intensity aerobic exercise have been shown to enhance attention, executive functioning, and processing speed through mechanisms such as increased cerebral blood flow, neurotrophic factor release, and elevated arousal [1,2,8,9,10,11]. However, the majority of this research has focused on structured aerobic or resistance training, with less attention paid to low-intensity, accessible modalities such as stretching. Stretching is likely to induce transient physiological responses such as changes in arousal or sensory input that can influence short-term cognitive performance.

Stretching, a low-intensity form of physical activity, is widely practiced for its musculoskeletal benefits, including improved flexibility, reduced muscle stiffness, and increased joint range of motion [12,13]. Unlike high-intensity exercise, stretching poses minimal risk, is easy to implement, and is well-tolerated across age groups [14,15,16]. Therefore, given that patients with physical impairments often have limited tolerance for high-intensity exercise, stretching offers a feasible and safe alternative that may simultaneously promote physical health and cognitive function [17].

While existing research has shown that acute physical activity can enhance certain aspects of cognitive performance, very few studies have investigated whether stretching can elicit similar effects [9,11,17]. Moreover, among the limited studies available, cognitive assessments have typically been conducted only immediately following the intervention, with little consideration for how long such benefits may last [8]. Thus, a clear gap remains in understanding the temporal dynamics, that is, the onset and duration, of cognitive improvements following self-stretching. Addressing this knowledge gap is important not only for healthy populations but also for clinical groups such as individuals with stroke, where rehabilitation strategies that are both effective and sustainable are urgently needed.

Therefore, the purpose of this study was to examine the time-dependent effects of self-stretching on cognitive function. We evaluated changes in attention, working memory, and short-term memory using three standardized behavioral tasks, the Stroop test, the N-back task, and the digit span-based memory test, administered at baseline and 10, 20, and 30 min post-intervention using a self-control design. Through this approach, we aimed to determine whether stretching produces measurable cognitive benefits and whether such benefits are sustained over time.

## 2. Methods

### 2.1. Subjects

Thirty healthy subjects, free from musculoskeletal and nervous system diseases, participated in the present study. The study utilized a self-control (within-subject) design where each participant’s post-intervention performance was compared against their own baseline. To investigate the time-course effects and mitigate learning effects from repeated cognitive testing, participants were randomly assigned to one of three groups using a computer-generated random sequence. The researchers collecting the data were not involved in assigning participants to the post-intervention time-point groups. Group 1 completed cognitive testing 10 min post-stretching, Group 2 was assessed 20 min post-stretching, and Group 3 underwent testing 30 min post-stretching. This design allowed for the assessment of time-dependent changes while ensuring that no single participant completed the full cognitive test set more than twice (Baseline and one post-intervention time point).

Participant demographics are summarized in Table 1. The following inclusion criteria were adopted: (1) the absence of any lesion that might have affected the experiment; (2) the ability to understand and follow the experiment; (3) inactive (<2 days per week of structured physical activity) for at least the previous 1 month; (4) no cognitive deficiency. The study was explained in detail to all participants before commencement, and the consent form for participation was distributed and signed by all prior to their involvement in the experiment.

### 2.2. Self-Stretching Protocol

We selected the cervical and shoulder self-stretching protocol for two reasons. First, this low-intensity exercise can be easily performed without constraints of time or space, thus enhancing the generalizability of our findings. Furthermore, stretching these specific muscles (e.g., upper trapezius, levator scapulae) provides highly potent afferent sensory input crucial for modulating the central nervous system’s spatial awareness and attentional networks [18,19].

Participants performed bilateral static self-stretching targeting four specific muscles: the suboccipital, levator scapulae, sternocleidomastoid (SCM), and upper trapezius. For each muscle, three repetitions were completed per side, with each stretch held for 15 s, followed by a 10 s rest interval. All stretching exercises were conducted in a seated position, with participants instructed to maintain an elongated cervical posture throughout [20]. The specific stretching procedures for each muscle are described as follows:

SCM Stretch: Participants rotated their head maximally to the opposite side within a pain-free range to elongate the SCM. Using the contralateral hand placed above the ear, they applied a gentle stretch at the end range without inducing pain. Upper Trapezius Stretch: The ipsilateral hand was placed behind the back to stabilize the scapula. Participants rotated their head toward the target side, side-bent away from it, and flexed the neck forward. The contralateral hand was used to gently guide the head into the stretch position. Levator Scapulae Stretch: With the head side-bent and rotated away from the target side, participants reached downward and backward with the target-side hand to anchor the scapula by holding the chair. The opposite hand was placed on the head to apply a controlled stretch diagonally forward and laterally. Suboccipital Stretch: Participants first performed a chin tuck to achieve axial elongation, then nodded the head forward by bringing the chin toward the larynx until a stretch sensation was felt in the suboccipital region. This standardized stretching protocol was self-administered under supervision to ensure proper technique and safety.

### 2.3. Cognitive Function Assessment

#### 2.3.1. Stroop Test

To assess selective attention, the Stroop test was administered using the color-word (CW) condition, which is freely available online (http://psychtoolbox.org/ accessed on 13 December 2024). Prior to the test, participants were provided with standardized instructions and were given one minute to practice the task to ensure task familiarity [21].

In the CW condition, color words (e.g., “red,” “green”) were presented in incongruent ink colors (e.g., the word “red” printed in green ink). Participants were instructed to name the ink color, not the written word, as quickly and accurately as possible. This condition requires the inhibition of the automatic word-reading response in favor of a less automated task, color identification, thereby engaging executive control processes. Cognitive performance on the Stroop test was evaluated based on reaction time (ms) and accuracy, with a maximum score of 40.

#### 2.3.2. N-Back Test

The N-back task, available through the Psychtoolbox platform (http://psychtoolbox.org/ accessed on 13 December 2024), was used to evaluate working memory performance [22]. In this study, a 2-back condition was implemented, in which participants were presented with a continuous sequence of visual stimuli and were required to identify whether the current item matched the one shown two positions earlier.

The task outcome was assessed based on accuracy (percentage of correct detections) and the number of errors (incorrect responses). A higher accuracy rate and fewer errors indicated better working memory function. All participants completed the same sequence of stimuli under standardized testing conditions. Prior to the main task, a brief practice session was provided to ensure understanding of the task requirements.

#### 2.3.3. Digit Span-Based Memory Test

The digit span-based memory test [23] was assessed to evaluate short-term memory capacity, which is freely available online at https://metodorf.com/tests/memory/memorize_rows_numbers.php (accessed on 13 December 2024). The task involved progressively increasing digit sequences, starting from 5 digits and extending to 20 digits. In this task, participants were shown a sequence of numerical stimuli arranged in rows [22]. They were instructed to respond as accurately as possible when recognizing a previously presented number row. The task measured maximum memory span, defined as the highest number of consecutive correct recognitions, and total accuracy score. All participants were given a standardized one-minute practice session before the formal assessment to ensure task familiarity.

### 2.4. Experimental Procedure

Participants underwent three tests: The Stroop test, the N-back test, and the Digit span-based memory test, as a baseline assessment on the first day. On the second day, participants received education on self-stretching exercises and performed the exercises themselves. After the self-stretching session, participants underwent the three tests again. The order of the three tests was randomized. To minimize the learning effect, there was at least a 120 h period between the first day and the second day.

It should be noted that participants were assigned to only one of the three post-intervention time points to prevent learning effects from repeated cognitive testing. Although participants were assigned to different post-intervention time points, each participant was compared only with their own baseline value. The study was conducted in accordance with the Declaration of Helsinki and approved by the Institutional Review Board of Uiduk University (Approval No. 1041553-202409-009-02).

### 2.5. Data Analysis

The Shapiro–Wilk test was used to assess the normal distribution of the participants. Paired t-tests were performed to assess differences between baseline and post-stretching measurements at 10, 20, and 30 min. Standardized mean changes were computed as Hedges’ g with 95% confidence intervals (CIs). To mitigate the limitation of the self-controlled design, one-way ANOVAs were conducted on baseline measures to confirm homogeneity, thereby supporting the validity of the within-subject design. For data not satisfying the normality assumption, the Wilcoxon signed-rank test was applied as a non-parametric alternative. The non-normal variables included: (1) Stroop scores at baseline and post-intervention across all time points (10, 20, and 30 min), (2) post-intervention N-back accuracy at 20 and 30 min and all N-back error indices (10, 20, and 30 min), and (3) baseline span and baseline total memory scores (10 and 20 min). These statistical analyses were conducted using IBM SPSS Statistics 22 (IBM Corp., Armonk, NY, USA). Statistical significance was accepted for *p*-values less than 0.05.

## 3. Results

### 3.1. Stroop Test Results

A total of three groups were assessed at baseline and at 10, 20, and 30 min post-stretching (Table 2). Stroop scores, which represent accuracy, showed no significant changes at any of the post-stretching time points. The effect size was small, indicating a limited magnitude of change.

Reaction time showed a significant decrease at 10 and 20 min post-stretching. No significant difference was observed at 30 min (*p* = 0.105). The effect size for the decrease in reaction time after stretching was large at 10 (g = −1.01) and 20 (g = −0.87) minutes and moderate at 30 (g = −0.52) minutes.

### 3.2. N-Back Test Results

Performance on the N-back task did not show any significant changes at 10, 20, or 30 min after stretching (Table 3). Although accuracy slightly improved and error rates decreased in the early time points, these differences were not statistically significant (*p* > 0.05). The corresponding effect sizes were small, indicating minimal effects on working memory.

### 3.3. Memory Test Results

Table 4 shows that no significant changes were found in either maximum memory span or total score at any of the measured time points (*p* > 0.05). The effect sizes were small to moderate, indicating minimal effects of self-stretching on short-term memory performance.

### 3.4. Baseline Comparisons

One-way ANOVA showed no significant baseline differences among the three groups in any of the cognitive measures (*p* > 0.05), confirming homogeneity prior to the stretching intervention (Table 5).

## 4. Discussion

The present study investigated the time-course effects of self-stretching on cognitive performance, specifically focusing on attention (Stroop test), working memory (N-back test), and short-term memory (digit span-based memory test). While self-stretching did not produce statistically significant improvements in overall test scores, notable short-term effects were observed in response times, particularly within the Stroop test.

A significant reduction in Stroop reaction time, accompanied by a large effect size, was observed at 10 and 20 min post-stretching, indicating that stretching may provide transient benefits in information-processing speed. However, this effect was not maintained at 30 min, where a moderate effect size was observed but did not reach statistical significance, suggesting that the cognitive benefit is short-lived and diminishes with time. Sudo et al. reported that a 10 min stretching session was directly associated with faster Stroop reaction times [17]. Barella et al. found that a bout of acute exercise led to improvements in reaction-time–based cognitive performance [8]. Additionally, large-scale evidence further demonstrated that low to moderate intensity physical activity produced modest but reliable improvements in reaction time-based executive functions, whereas accuracy or memory-related outcomes showed smaller or inconsistent changes [18]. These findings align with our research, suggesting that the increases in cognitive performance are driven by changes in state processes, rather than computational processes [8,9,17,21]. A reasonable explanation for these findings is that self-stretching may lead to transient increases in blood flow and brief increases in arousal, thereby enhancing attentional processing [8,9,11,24].

In contrast, no significant improvements were found with small effect sizes in the accuracy or performance scores of the N-back and Digit span-based memory tests. These results suggest that self-stretching may not be sufficient to influence higher-order cognitive functions such as working memory accuracy or retention capacity. This contrasts with studies that have reported broader cognitive gains following moderate-intensity aerobic exercise [25], highlighting the possibility that intensity, duration, or modality of exercise plays a critical role in determining cognitive outcomes. One possible explanation for the selective improvement in reaction time but not accuracy may be the nature of the stretching intervention, which might activate arousal or attentional systems without sufficiently engaging the neural circuits required for memory or executive function improvements [8]. Furthermore, the absence of significant findings in N-back and memory tests may reflect ceiling effects in a healthy young adult sample or limited task sensitivity.

This study also provides insight into the duration of cognitive effects [8]. The beneficial effect on reaction time observed at 10 and 20 min appears to diminish by 30 min, supporting the hypothesis that cognitive enhancement from acute physical stimulation is transient. These findings reinforce previous conclusions that improvements in speed of processing after acute exercise are driven by temporary physiological and neurochemical changes (e.g., increased cerebral blood flow, arousal level) rather than structural adaptation [8,9]. For rehabilitation purposes, this suggests that stretching-based interventions might need to be delivered repeatedly or integrated with task-specific training to sustain benefits across therapy sessions.

Several limitations must be noted. First, the sample size in each group may have been underpowered to detect small to moderate effects in memory or N-back performance. Second, because this study included only young, healthy adults, the findings may not generalize to older individuals or clinical populations. Third, the lack of a non-intervention control group may limit the strength of causal inferences. Although a non-intervention control group was not included, the large effect sizes observed at 10 and 20 min strengthen the internal validity of the findings by indicating consistent and meaningful within-subject effects. Given the primary aim of exploring short-term, time-dependent changes in cognitive performance, a self-control design was considered appropriate for minimizing inter-individual variability while capturing within-subject responses. Moreover, baseline ANOVA confirmed group homogeneity prior to the intervention, supporting the reliability of the within-subject design despite the absence of a separate control group. Nevertheless, future studies incorporating randomized controlled or crossover designs, with sham conditions, are needed to confirm these preliminary findings. Finally, participant motivation, task familiarity, or inter-individual variability in fitness level and cognitive baseline may have influenced the results.

## 5. Conclusions

Self-stretching appears to provide short-term cognitive benefits, particularly in enhancing processing speed as measured by reaction time in attention tasks, and these effects seem to persist for up to 20 min post-intervention. However, these effects are brief and task-specific, with no observable impact on memory or working memory accuracy. These results suggest that self-stretching may offer limited but potentially useful short-term support for attentional processing, serving as a low-intensity supplementary strategy in situations that require quick cognitive responses. Future studies should explore the optimal timing, frequency, and intensity of stretching-based interventions and investigate their impact across broader age groups and populations with cognitive impairments.

## Figures and Tables

**Table 1:** General Characteristics of Subjects.

Number of Subjects	30	
Age	20.77 (1.04)	
Gender	Male: 15	Female: 15
Height (cm)	176.22 (5.11)	162.47 (4.44)
Weight (kg)	81.53 (20.28)	58.06 (8.28)
BMI	26.12 (5.73)	22.01 (3.04)
Education (year)	13.59 (0.63)	

Values represent mean (±standard deviation).

**Table 2:** Changes in Stroop Test Performance After Self-Stretching.

Group	Measure	Time Point	Mean (±SD)	*p* Value	Hedges’ g	95% CI
Group 1	Score	Baseline	33.9 (10.54)	0.28	0.25	[−0.35, 0.84]
		After 10 m	35.9 (9.16)			
	Response time (ms)	Baseline	976.89 (171.04)	0.01 *	−1.01	[−1.87, −0.16]
		After 10 m	808.02 (124.69)			
Group 2	Score	Baseline	37.3 (2.06)	0.16	0.18	[−0.43, 0.78]
		After 20 m	38.7 (1.64)			
	Response time (ms)	Baseline	853.63 (205.26)	0.02 *	−0.87	[−1.67, −0.06]
		After 20 m	722.97 (126.02)			
Group 3	Score	Baseline	38.4 (1.71)	0.56	0.08	[−0.48, 0.65]
		After 30 m	38.8 (1.40)			
	Response time (ms)	Baseline	905.38 (272.99)	0.11	−0.52	[−1.23, 0.19]
		After 30 m	809.25 (176.73)			

SD: standard deviation, m: minute, ms: millisecond, CI: Confidence Interval. * There is a significant difference between baseline and each post-stretching time point. (*p* < 0.05).

**Table 3:** Changes in N-back Task Performance After Self-Stretching.

Group	Measure	Time Point	Mean (±SD)	*p* Value	Hedges’ g	95% CI
Group 1	Accuracy (%)	Baseline	73 (25.09)	0.68	0.13	[−0.48, 0.74]
		After 10 m	76.2 (18.90)			
	Error rate (%)	Baseline	4.8 (5.05)	0.53	−0.17	[−0.80, 0.46]
		After 10 m	3.1 (5.34)			
Group 2	Accuracy (%)	Baseline	69.3 (13.82)	0.65	0.19	[−0.45, 0.83]
		After 20 m	72.6 (13.34)			
	Error rate (%)	Baseline	1 (3.16)	0.68	−0.11	[−0.73, 0.52]
		After 20 m	0.5 (1.58)			
Group 3	Accuracy (%)	Baseline	75.1 (22.78)	0.81	−0.08	[−0.71, 0.54]
		After 30 m	73.3 (24.87)			
	Error rate (%)	Baseline	6.6 (7.12)	0.22	−0.31	[−1.97, 0.35]
		After 30 m	4.1 (4.79)			

SD: standard deviation, CI: Confidence Interval.

**Table 4:** Changes in Digit-Span Based Memory Test After Self-Stretching.

Group	Measure	Time Point	Mean (±SD)	*p* Value	Hedges’ g	95% CI
Group 1	Maximum memory span (n)	Baseline	9.3 (0.82)	0.51	0.21	[−0.39, 0.81]
		After 10 m	9.5 (1.08)			
	Score	Baseline	59.4 (6.11)	0.14	0.47	[−1.10, 0.16]
		After 10 m	54.7 (6.77)			
Group 2	Maximum memory span (n)	Baseline	8.3 (1.16)	0.31	0.35	[−0.29, 0.99]
		After 20 m	8.7 (1.16)			
	Score	Baseline	54.6 (8.81)	0.82	0.07	[−0.56, 0.69]
		After 20 m	55.2 (2.86)			
Group 3	Maximum memory span (n)	Baseline	8.8 (1.32)	0.56	0.25	[−0.38, 0.88]
		After 30 m	9.1 (1.29)			
	Score	Baseline	51.3 (3.48)	0.10	0.58	[−0.08, 1.23]
		After 30 m	58.3 (2.39)			

SD: standard deviation, CI: Confidence Interval.

**Table 5:** Baseline Characteristics and Between-Group Comparisons.

Task	Measure	Mean of Group 1	Mean of Group 2	Mean of Group 3	F Value	*p* Value
Stroop test	Score	33.90 (10.54)	37.30 (2.06)	38.40 (1.71)	1.838	0.179
	Response time (ms)	976.89 (171.04)	853.63 (205.26)	905.38 (272.99)	0.908	0.415
N-Back test	Accuracy (%)	73.00 (25.10)	69.30 (13.83)	75.10 (22.78)	0.237	0.791
	Error rate (%)	4.80 (5.05)	1.00 (3.16)	6.60 (7.12)	2.656	0.088
Digit-span based memory test	Maximum memory span (n)	9.30 (0.82)	8.30 (1.16)	8.80 (1.32)	1.706	0.201
	Accuracy (%)	0.57 (0.05)	0.53 (0.08)	0.54 (0.08)	1.139	0.334

Values represent mean (±standard deviation), ms: millisecond.

## Data Availability

The data supporting the findings of this study are available from the corresponding author upon reasonable request. The data are not publicly available because of privacy and ethical re-strictions.
